# 1,3-Dicyclo­hexyl-3-[(pyridin-2-yl)carbon­yl]urea monohydrate from synchrotron radiation

**DOI:** 10.1107/S1600536811037512

**Published:** 2011-09-17

**Authors:** Alessandra C. Pinheiro, Marcus V. N. de Souza, James L. Wardell, Solange M. S. V. Wardell, Edward R. T. Tiekink

**Affiliations:** a Instituto de Tecnologia em Farmacos, Fundação Oswaldo Cruz (FIOCRUZ), FarManguinhos, Rua Sizenando Nabuco, 100, Manguinhos, 21041-250 Rio de Janeiro, RJ, Brazil; b Centro de Desenvolvimento Tecnológico em Saúde (CDTS), Fundação Oswaldo Cruz (FIOCRUZ), Casa Amarela, Campus de Manguinhos, Av. Brasil 4365, 21040-900 Rio de Janeiro, RJ, Brazil; c CHEMSOL, 1 Harcourt Road, Aberdeen AB15 5NY, Scotland; d Department of Chemistry, University of Malaya, 50603 Kuala Lumpur, Malaysia

## Abstract

The title urea derivative crystallizes as a monohydrate, C_19_H_27_N_3_O_2_·H_2_O. The central C_3_N grouping is almost planar (r.m.s. deviation = 0.0092 Å), and the amide and pyridine groups are substanti­ally twisted out this plane [dihedral angles = 62.80 (12) and 34.98 (10)°, respectively]. Supra­molecular double chains propagating along the *b*-axis direction feature in the crystal packing whereby linear chains sustained by N—H⋯O hydrogen bonds formed between the amide groups are linked by helical chains of water mol­ecules (linked by O—H⋯O hydrogen bonds). The H atom that participates in these water chains is disordered over two positions of equal occupancy. The double chains are connected into a two-dimensional array by C—H⋯O contacts and the layers stack along the *a* axis.

## Related literature

For the preparation of *N*-(arenecarbon­yl)-*N*,*N*′-dicyclo­hexyl­urea derivatives, see: Kaiser *et al.* (2008 ▶); Neves Filho *et al.* (2007 ▶); Schotman (1991 ▶). For the crystal structures of related *N*-(arenecarbon­yl)-*N*,*N*′-dicyclo­hexyl­urea derivatives, see: Chérioux *et al.* (2002 ▶); Cai *et al.* (2009 ▶); Dhinaa *et al.* (2010 ▶); Orea Flores *et al.* (2006 ▶); Gallagher *et al.* (1999 ▶); Wang & Zhou (2008 ▶); Wu *et al.* (2006 ▶).
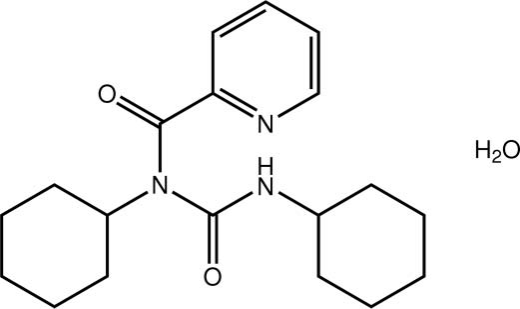


## Experimental

### 

#### Crystal data

C_19_H_27_N_3_O_2_·H_2_O
*M* *_r_* = 347.46
Monoclinic, 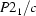

*a* = 18.639 (19) Å
*b* = 5.035 (5) Å
*c* = 21.59 (2) Å
β = 111.395 (9)°
*V* = 1887 (3) Å^3^
*Z* = 4
Synchrotron radiation
λ = 0.6905 Å
μ = 0.05 mm^−1^
*T* = 120 K
0.25 × 0.08 × 0.02 mm

#### Data collection

Bruker SMART APEXII CCD diffractometer
Absorption correction: multi-scan (*SADABS*; Sheldrick, 2007 ▶) *T*_min_ = 0.743, *T*_max_ = 1.000
13440 measured reflections
3810 independent reflections
3200 reflections with *I* > 2σ(*I*)
*R*_int_ = 0.043

#### Refinement

*R*[*F* ^2^ > 2σ(*F* ^2^)] = 0.048
*wR*(*F* ^2^) = 0.126
*S* = 1.06
3810 reflections
238 parameters
7 restraints
H atoms treated by a mixture of independent and constrained refinement
Δρ_max_ = 0.36 e Å^−3^
Δρ_min_ = −0.20 e Å^−3^

### 

Data collection: *APEX2* (Bruker, 2004 ▶); cell refinement: *SAINT* (Bruker, 2004 ▶); data reduction: *SAINT*; program(s) used to solve structure: *SHELXS97* (Sheldrick, 2008 ▶); program(s) used to refine structure: *SHELXL97* (Sheldrick, 2008 ▶); molecular graphics: *ORTEP-3* (Farrugia, 1997 ▶) and *DIAMOND* (Brandenburg, 2006 ▶); software used to prepare material for publication: *publCIF* (Westrip, 2010 ▶).

## Supplementary Material

Crystal structure: contains datablock(s) global, I. DOI: 10.1107/S1600536811037512/hb6408sup1.cif

Structure factors: contains datablock(s) I. DOI: 10.1107/S1600536811037512/hb6408Isup2.hkl

Supplementary material file. DOI: 10.1107/S1600536811037512/hb6408Isup3.cml

Additional supplementary materials:  crystallographic information; 3D view; checkCIF report

## Figures and Tables

**Table 1:** Hydrogen-bond geometry (Å, °)

*D*—H⋯*A*	*D*—H	H⋯*A*	*D*⋯*A*	*D*—H⋯*A*
N1—H1n⋯O1^i^	0.88 (1)	2.07 (1)	2.908 (3)	160 (2)
O1w—H1w⋯O2	0.84 (2)	1.98 (2)	2.820 (3)	174 (2)
O1w—H2w⋯O1w^ii^	0.84 (3)	1.97 (3)	2.773 (4)	162 (4)
O1w—H3w⋯O1w^iii^	0.84 (3)	1.98 (3)	2.799 (4)	167 (4)
C17—H17⋯O1w^iv^	0.95	2.59	3.517 (4)	164
C18—H18⋯O2^v^	0.95	2.47	3.367 (4)	157

Symmetry codes: (i) 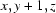; (ii) 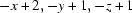; (iii) 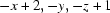; (iv) 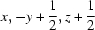; (v) 
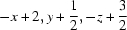
.

**Fig. 1.**
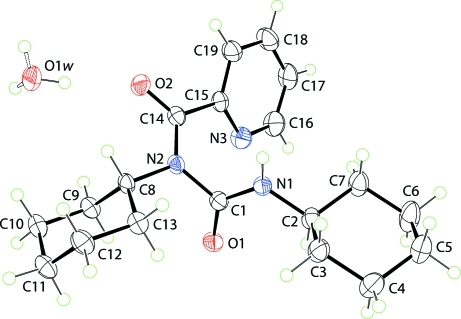
The molecular structure of (I) showing displacement ellipsoids at the 50% probability level.

**Fig. 2.**
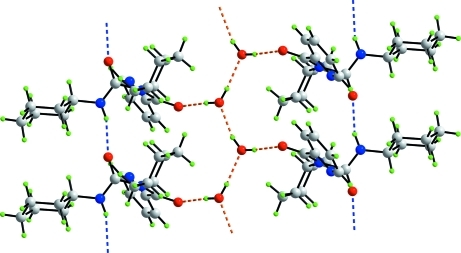
The supramolecular double-chain aligned along the b axis in the crystal structure of (I) formed through the agency of intermolecular O—H···O and N—H···O hydrogen bonding interactions shown as orange and blue dashed lines, respectively. Only one of the disordered water-H atoms is represented.

**Fig. 3.**
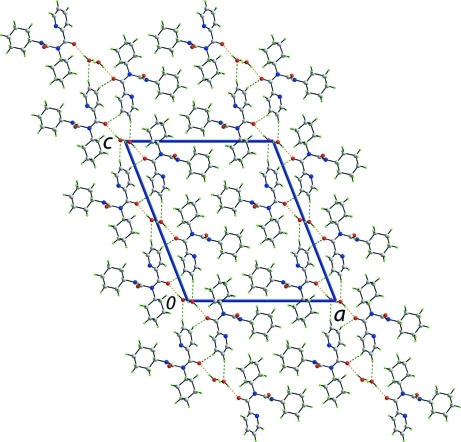
A view of the unit-cell contents in (I) shown in projection down the b axis [the direction of the supramolecular chains illustrated in Fig. 2] and highlighting the stacking of layers along the a direction. The O—H···O hydrogen bonds and C—H···O interactions are shown as orange and green dashed lines, respectively

## supplementary crystallographic information

### Comment

Reactions of arenecarboxylic acids with dicyclohexylcarbodiimide (DCC) in the presence of a catalyst, such as 1-hydroxybenzotriazole, HOBt, produce *N*-(arenecarbonyl)-*N*,*N*'-dicyclohexylureas (Kaiser *et al.*, 2008; Neves Filho *et al.*, 2007; Schotman, 1991). Several crystal structures of *N*-(arenecarbonyl)-*N*,*N*'-dicyclohexylurea derivatives have been reported (Chérioux *et al.*, 2002; Cai *et al.*, 2009; Dhinaa *et al.*, 2010; Orea Flores *et al.*, 2006; Gallagher *et al.*, 1999; Wang *et al.*, 2008; Wu *et al.*, 2006). Herein, we now report the crystal structure of the monohydrate of *N*,*N*'-dicyclohexyl- *N*-(pyridine-2-carbonyl)urea, (I).

A molecule of *N*,*N*'-dicyclohexyl- *N*-(pyridine-2-carbonyl)urea and a water molecule of solvation comprise the asymmetric unit of (I). Two of the water bound H atoms are disordered and have been assigned site occupancy factors of 0.50. The disorder is accounted for in terms of the dictates of hydrogen bonding in the crystal structure, see below. The pyridine ring is twisted out of the central C_3_N plane (r.m.s. deviation = 0.0092 Å) with the dihedral angle being 34.98 (10) °. The amide group is even more twisted out of the plane through the central ring forming a dihedral angle of 62.80 (12) °. Each of the cyclohexyl rings adopts a chair conformation.

Hydrogen bonding of the type O—H···O and N—H···O lead to the formation of supramolecular chains along the *b* axis, Table 1. The amide groups self-associate to form linear chains. Pairs of chains are linked by hydrogen bonding interactions involving the water molecules. Thus, the carbonyl-O2 atom is linked to the water molecule, and the remaining water-H atoms (each with site occupancy factor = 1/2) link water molecules into a helical chain, Fig. 2. The chains are linked into layers *via* C—H···O interactions, Table 1, which stack along the *a* direction.

### Experimental

To a stirred solution of the pyridin-2-carboxylic acid (1 mmol) in anhydrous CH_2_Cl_2_ (25 ml) were added DCC (0.8 mmol, 1 equiv.) and HOBt (*ca* 10 mg). After leaving at room temperature for 2 h, the precipitate of *N*,*N*'-dicyclohexylurea was removed and the filtrate was poured into saturated aqueous NaHCO_3_ solution (20 ml). The organic material was extracted into EtOAc (3 *x* 20 ml), the combined layers dried over MgSO_4_, and rotary evaporated. The residue was chromatographed (10% to 50% EtOAc/hexanes) to give *N*-(pyridin-2-carbonyl)-*N*,*N*'-dicyclohexylurea. Yield: 60%, as a white solid.  Recrystallization from moist EtOH gave the monohydrate as colourless laths.

^1^H NMR (400 MHz, CDCl_3_) δ: 8.57 (d, J = 3.6, 1H, H6), 7.78 (m, 1H, H4), 7.68 (d, J = 7.6, 1H, H3), 7.37 (m, 1H, H5), 6.09 (s, 1H, NH), 4.20 (m, 1H, NCH), 3.51 (m, 1H, NHCH), 0.8–2 (m, 20 H, cyclohexyl) p.p.m.. ^13^C NMR (100 MHz, CDCl_3_) δ: 168.1 (CON), 154.0 (CONH), 153.9 (C2), 148.5 (C6), 137.0 (C4), 125.1, 122.9 (C3 and C5), 56.4 (NCH), 49.8 (NHCH), 33.9, 32.4, 30.7, 26.2, 25.6, 25.4, 25.3, 24.9, 24.6 (cyclohexyl) p.p.m.. *M*.pt.: 415 K. IR (cm^-1^; KBr): 1710 (CONH) and 1680 (CON).

### Refinement

The C-bound H atoms were geometrically placed (C–H = 0.95–1.00 Å) and refined as riding with *U*_iso_(H) = 1.2*U_eq_*(parent atom). The O– and N-bound H atom were refined with the distance restraints 0.84±0.01 and 0.88±0.01 Å, respectively, and with *U*_iso_(H) = *yU*_eq_(parent atom) for *y* = 1.2 for N and *y* = 1.5 for O. One of the water-bound H atoms was found to be disordered over two positions and each was assigned a site occupancy factor = 0.50.

### Crystal data

**Table:**

C_19_H_27_N_3_O_2_·H_2_O	*F*(000) = 752
*M**_r_* = 347.46	*D*_x_ = 1.223 Mg m^−^^3^
Monoclinic, *P*2_1_/*c*	Synchrotron radiation, λ = 0.6905 Å
Hall symbol: -P 2ybc	Cell parameters from 908 reflections
*a* = 18.639 (19) Å	θ = 4.6–25.5°
*b* = 5.035 (5) Å	µ = 0.05 mm^−^^1^
*c* = 21.59 (2) Å	*T* = 120 K
β = 111.395 (9)°	Lath, colourless
*V* = 1887 (3) Å^3^	0.25 × 0.08 × 0.02 mm
*Z* = 4	

### Data collection

**Table:**

Bruker SMART APEXII CCD diffractometer	3810 independent reflections
Radiation source: Daresbury SRS station 9.8	3200 reflections with *I* > 2σ(*I*)
silicon 111	*R*_int_ = 0.043
fine–slice ω scans	θ_max_ = 25.6°, θ_min_ = 3.3°
Absorption correction: multi-scan (*SADABS*; Sheldrick, 2007)	*h* = −22→23
*T*_min_ = 0.743, *T*_max_ = 1.000	*k* = −6→6
13440 measured reflections	*l* = −25→26

### Refinement

**Table:**

Refinement on *F*^2^	Primary atom site location: structure-invariant direct methods
Least-squares matrix: full	Secondary atom site location: difference Fourier map
*R*[*F*^2^ > 2σ(*F*^2^)] = 0.048	Hydrogen site location: inferred from neighbouring sites
*wR*(*F*^2^) = 0.126	H atoms treated by a mixture of independent and constrained refinement
*S* = 1.06	*w* = 1/[σ^2^(*F*_o_^2^) + (0.0475*P*)^2^ + 0.9558*P*] where *P* = (*F*_o_^2^ + 2*F*_c_^2^)/3
3810 reflections	(Δ/σ)_max_ < 0.001
238 parameters	Δρ_max_ = 0.36 e Å^−^^3^
7 restraints	Δρ_min_ = −0.20 e Å^−^^3^

### Special details

**Table:**

Geometry. All s.u.'s (except the s.u. in the dihedral angle between two l.s. planes) are estimated using the full covariance matrix. The cell s.u.'s are taken into account individually in the estimation of s.u.'s in distances, angles and torsion angles; correlations between s.u.'s in cell parameters are only used when they are defined by crystal symmetry. An approximate (isotropic) treatment of cell s.u.'s is used for estimating s.u.'s involving l.s. planes.
Refinement. Refinement of *F*^2^ against ALL reflections. The weighted *R*-factor *wR* and goodness of fit *S* are based on *F*^2^, conventional *R*-factors *R* are based on *F*, with *F* set to zero for negative *F*^2^. The threshold expression of *F*^2^ > 2σ(*F*^2^) is used only for calculating *R*-factors(gt) *etc*. and is not relevant to the choice of reflections for refinement. *R*-factors based on *F*^2^ are statistically about twice as large as those based on *F*, and *R*- factors based on ALL data will be even larger.

### Fractional atomic coordinates and isotropic or equivalent isotropic displacement parameters (Å^2^)

**Table:**

	*x*	*y*	*z*	*U*_iso_*/*U*_eq_	Occ. (<1)
O1	0.70258 (7)	−0.1731 (2)	0.59067 (6)	0.0303 (3)	
O2	0.91282 (6)	0.2990 (2)	0.60869 (5)	0.0301 (3)	
N1	0.69415 (7)	0.2624 (3)	0.61576 (7)	0.0243 (3)	
H1N	0.7086 (10)	0.425 (2)	0.6115 (9)	0.029*	
N2	0.79274 (7)	0.1277 (3)	0.58142 (6)	0.0234 (3)	
N3	0.83745 (8)	0.0413 (3)	0.72128 (7)	0.0337 (3)	
C1	0.72692 (9)	0.0548 (3)	0.59806 (7)	0.0240 (3)	
C2	0.62543 (9)	0.2342 (3)	0.63269 (8)	0.0258 (3)	
H2	0.6196	0.0428	0.6423	0.031*	
C3	0.55414 (10)	0.3205 (4)	0.57442 (8)	0.0338 (4)	
H3A	0.5482	0.2074	0.5354	0.041*	
H3B	0.5606	0.5065	0.5625	0.041*	
C4	0.48146 (10)	0.2991 (4)	0.59151 (9)	0.0377 (4)	
H4A	0.4366	0.3659	0.5537	0.045*	
H4B	0.4719	0.1105	0.5988	0.045*	
C5	0.49011 (10)	0.4583 (4)	0.65350 (9)	0.0396 (4)	
H5A	0.4935	0.6496	0.6443	0.048*	
H5B	0.4440	0.4314	0.6653	0.048*	
C6	0.56154 (11)	0.3759 (5)	0.71165 (9)	0.0468 (5)	
H6A	0.5553	0.1908	0.7243	0.056*	
H6B	0.5673	0.4914	0.7503	0.056*	
C7	0.63413 (10)	0.3957 (4)	0.69461 (8)	0.0354 (4)	
H7A	0.6438	0.5840	0.6870	0.043*	
H7B	0.6790	0.3294	0.7325	0.043*	
C8	0.78688 (9)	0.0807 (3)	0.51160 (7)	0.0245 (3)	
H8	0.8211	0.2131	0.5015	0.029*	
C9	0.81573 (9)	−0.1941 (3)	0.50312 (8)	0.0276 (3)	
H9A	0.7841	−0.3306	0.5141	0.033*	
H9B	0.8697	−0.2155	0.5341	0.033*	
C10	0.81124 (10)	−0.2337 (3)	0.43150 (8)	0.0308 (4)	
H10A	0.8470	−0.1091	0.4221	0.037*	
H10B	0.8274	−0.4169	0.4261	0.037*	
C11	0.72974 (10)	−0.1862 (4)	0.38230 (8)	0.0333 (4)	
H11A	0.6947	−0.3211	0.3891	0.040*	
H11B	0.7287	−0.2052	0.3363	0.040*	
C12	0.70189 (10)	0.0901 (4)	0.39131 (8)	0.0331 (4)	
H12A	0.6483	0.1150	0.3599	0.040*	
H12B	0.7346	0.2253	0.3812	0.040*	
C13	0.70517 (9)	0.1285 (3)	0.46261 (8)	0.0285 (3)	
H13A	0.6889	0.3113	0.4681	0.034*	
H13B	0.6694	0.0028	0.4717	0.034*	
C14	0.85976 (9)	0.2239 (3)	0.62575 (7)	0.0241 (3)	
C15	0.87147 (9)	0.2303 (3)	0.69852 (7)	0.0250 (3)	
C16	0.85324 (11)	0.0380 (4)	0.78661 (9)	0.0406 (4)	
H16	0.8300	−0.0960	0.8039	0.049*	
C17	0.90154 (11)	0.2181 (4)	0.83078 (8)	0.0396 (4)	
H17	0.9112	0.2064	0.8770	0.048*	
C18	0.93532 (11)	0.4146 (4)	0.80637 (9)	0.0408 (4)	
H18	0.9679	0.5435	0.8352	0.049*	
C19	0.92055 (10)	0.4198 (4)	0.73843 (8)	0.0342 (4)	
H19	0.9436	0.5503	0.7199	0.041*	
O1W	0.97088 (8)	0.2514 (3)	0.50550 (6)	0.0385 (3)	
H1W	0.9509 (12)	0.257 (5)	0.5347 (9)	0.058*	
H2W	0.998 (2)	0.385 (5)	0.507 (2)	0.058*	0.50
H3W	0.994 (2)	0.107 (5)	0.508 (2)	0.058*	0.50

### Atomic displacement parameters (Å^2^)

**Table:**

	*U*^11^	*U*^22^	*U*^33^	*U*^12^	*U*^13^	*U*^23^
O1	0.0384 (6)	0.0207 (6)	0.0363 (6)	−0.0051 (5)	0.0192 (5)	−0.0030 (5)
O2	0.0307 (6)	0.0385 (7)	0.0230 (5)	−0.0049 (5)	0.0121 (5)	0.0002 (5)
N1	0.0300 (7)	0.0190 (6)	0.0281 (7)	−0.0023 (5)	0.0155 (6)	−0.0002 (5)
N2	0.0295 (7)	0.0234 (6)	0.0195 (6)	−0.0012 (5)	0.0116 (5)	−0.0010 (5)
N3	0.0417 (8)	0.0361 (8)	0.0248 (7)	−0.0032 (6)	0.0138 (6)	0.0041 (6)
C1	0.0287 (8)	0.0223 (8)	0.0220 (7)	−0.0013 (6)	0.0104 (6)	0.0004 (6)
C2	0.0305 (8)	0.0239 (8)	0.0284 (8)	−0.0001 (6)	0.0172 (7)	0.0030 (6)
C3	0.0312 (9)	0.0483 (10)	0.0232 (8)	−0.0035 (7)	0.0114 (7)	−0.0021 (7)
C4	0.0293 (9)	0.0512 (11)	0.0327 (9)	−0.0017 (8)	0.0115 (7)	0.0044 (8)
C5	0.0344 (9)	0.0495 (11)	0.0410 (10)	0.0074 (8)	0.0208 (8)	0.0028 (8)
C6	0.0408 (10)	0.0770 (15)	0.0279 (9)	0.0091 (10)	0.0190 (8)	0.0026 (9)
C7	0.0320 (9)	0.0524 (11)	0.0225 (8)	0.0037 (8)	0.0106 (7)	−0.0017 (8)
C8	0.0313 (8)	0.0277 (8)	0.0169 (7)	−0.0018 (6)	0.0116 (6)	−0.0017 (6)
C9	0.0322 (8)	0.0265 (8)	0.0229 (8)	0.0026 (6)	0.0087 (6)	−0.0008 (6)
C10	0.0396 (9)	0.0294 (9)	0.0261 (8)	0.0027 (7)	0.0152 (7)	−0.0037 (7)
C11	0.0419 (10)	0.0359 (9)	0.0199 (8)	−0.0003 (7)	0.0085 (7)	−0.0042 (7)
C12	0.0367 (9)	0.0354 (9)	0.0228 (8)	0.0031 (7)	0.0058 (7)	−0.0014 (7)
C13	0.0320 (8)	0.0275 (8)	0.0266 (8)	0.0016 (7)	0.0113 (7)	−0.0013 (6)
C14	0.0307 (8)	0.0217 (7)	0.0210 (7)	0.0015 (6)	0.0107 (6)	0.0015 (6)
C15	0.0292 (8)	0.0277 (8)	0.0195 (7)	0.0029 (6)	0.0105 (6)	0.0009 (6)
C16	0.0501 (11)	0.0479 (11)	0.0275 (9)	−0.0006 (9)	0.0187 (8)	0.0071 (8)
C17	0.0428 (10)	0.0571 (12)	0.0188 (8)	0.0073 (9)	0.0111 (7)	0.0022 (8)
C18	0.0412 (10)	0.0490 (11)	0.0269 (9)	−0.0013 (8)	0.0061 (8)	−0.0112 (8)
C19	0.0417 (10)	0.0357 (10)	0.0274 (8)	−0.0052 (7)	0.0152 (7)	−0.0043 (7)
O1W	0.0490 (8)	0.0421 (7)	0.0340 (7)	−0.0052 (6)	0.0263 (6)	−0.0011 (6)

### Geometric parameters (Å, °)

**Table:**

O1—C1	1.223 (2)	C8—C13	1.523 (2)
O2—C14	1.234 (2)	C8—H8	1.0000
N1—C1	1.335 (2)	C9—C10	1.531 (3)
N1—C2	1.461 (2)	C9—H9A	0.9900
N1—H1N	0.875 (9)	C9—H9B	0.9900
N2—C14	1.356 (2)	C10—C11	1.522 (3)
N2—C1	1.446 (2)	C10—H10A	0.9900
N2—C8	1.490 (2)	C10—H10B	0.9900
N3—C16	1.332 (3)	C11—C12	1.522 (3)
N3—C15	1.333 (2)	C11—H11A	0.9900
C2—C7	1.522 (3)	C11—H11B	0.9900
C2—C3	1.522 (3)	C12—C13	1.530 (3)
C2—H2	1.0000	C12—H12A	0.9900
C3—C4	1.532 (3)	C12—H12B	0.9900
C3—H3A	0.9900	C13—H13A	0.9900
C3—H3B	0.9900	C13—H13B	0.9900
C4—C5	1.517 (3)	C14—C15	1.506 (2)
C4—H4A	0.9900	C15—C19	1.384 (2)
C4—H4B	0.9900	C16—C17	1.384 (3)
C5—C6	1.517 (3)	C16—H16	0.9500
C5—H5A	0.9900	C17—C18	1.377 (3)
C5—H5B	0.9900	C17—H17	0.9500
C6—C7	1.529 (3)	C18—C19	1.391 (3)
C6—H6A	0.9900	C18—H18	0.9500
C6—H6B	0.9900	C19—H19	0.9500
C7—H7A	0.9900	O1W—H1W	0.842 (10)
C7—H7B	0.9900	O1W—H2W	0.839 (10)
C8—C9	1.520 (3)	O1W—H3W	0.838 (10)
			
C1—N1—C2	121.98 (13)	C8—C9—C10	110.41 (13)
C1—N1—H1N	120.7 (12)	C8—C9—H9A	109.6
C2—N1—H1N	116.7 (12)	C10—C9—H9A	109.6
C14—N2—C1	124.18 (14)	C8—C9—H9B	109.6
C14—N2—C8	118.65 (13)	C10—C9—H9B	109.6
C1—N2—C8	117.10 (12)	H9A—C9—H9B	108.1
C16—N3—C15	116.63 (16)	C11—C10—C9	110.95 (14)
O1—C1—N1	125.83 (15)	C11—C10—H10A	109.5
O1—C1—N2	120.91 (13)	C9—C10—H10A	109.4
N1—C1—N2	113.01 (14)	C11—C10—H10B	109.4
N1—C2—C7	110.20 (13)	C9—C10—H10B	109.5
N1—C2—C3	110.25 (14)	H10A—C10—H10B	108.0
C7—C2—C3	110.76 (14)	C10—C11—C12	110.77 (14)
N1—C2—H2	108.5	C10—C11—H11A	109.5
C7—C2—H2	108.5	C12—C11—H11A	109.5
C3—C2—H2	108.5	C10—C11—H11B	109.5
C2—C3—C4	111.27 (15)	C12—C11—H11B	109.5
C2—C3—H3A	109.4	H11A—C11—H11B	108.1
C4—C3—H3A	109.4	C11—C12—C13	110.71 (14)
C2—C3—H3B	109.4	C11—C12—H12A	109.5
C4—C3—H3B	109.4	C13—C12—H12A	109.5
H3A—C3—H3B	108.0	C11—C12—H12B	109.5
C5—C4—C3	110.89 (15)	C13—C12—H12B	109.5
C5—C4—H4A	109.5	H12A—C12—H12B	108.1
C3—C4—H4A	109.5	C8—C13—C12	109.96 (14)
C5—C4—H4B	109.5	C8—C13—H13A	109.7
C3—C4—H4B	109.5	C12—C13—H13A	109.7
H4A—C4—H4B	108.1	C8—C13—H13B	109.7
C6—C5—C4	111.37 (16)	C12—C13—H13B	109.7
C6—C5—H5A	109.4	H13A—C13—H13B	108.2
C4—C5—H5A	109.4	O2—C14—N2	122.07 (15)
C6—C5—H5B	109.4	O2—C14—C15	118.57 (14)
C4—C5—H5B	109.4	N2—C14—C15	119.32 (14)
H5A—C5—H5B	108.0	N3—C15—C19	123.94 (15)
C5—C6—C7	111.68 (16)	N3—C15—C14	117.52 (14)
C5—C6—H6A	109.3	C19—C15—C14	118.40 (14)
C7—C6—H6A	109.3	N3—C16—C17	123.96 (18)
C5—C6—H6B	109.3	N3—C16—H16	118.0
C7—C6—H6B	109.3	C17—C16—H16	118.0
H6A—C6—H6B	107.9	C18—C17—C16	118.71 (17)
C2—C7—C6	110.88 (15)	C18—C17—H17	120.6
C2—C7—H7A	109.5	C16—C17—H17	120.6
C6—C7—H7A	109.5	C17—C18—C19	118.37 (17)
C2—C7—H7B	109.5	C17—C18—H18	120.8
C6—C7—H7B	109.5	C19—C18—H18	120.8
H7A—C7—H7B	108.1	C15—C19—C18	118.38 (17)
N2—C8—C9	111.57 (12)	C15—C19—H19	120.8
N2—C8—C13	111.29 (13)	C18—C19—H19	120.8
C9—C8—C13	111.61 (13)	H1W—O1W—H2W	112 (3)
N2—C8—H8	107.4	H1W—O1W—H3W	109 (3)
C9—C8—H8	107.4	H2W—O1W—H3W	114 (4)
C13—C8—H8	107.4		
			
C2—N1—C1—O1	3.9 (2)	C8—C9—C10—C11	−55.90 (18)
C2—N1—C1—N2	178.19 (12)	C9—C10—C11—C12	56.71 (19)
C14—N2—C1—O1	−118.72 (17)	C10—C11—C12—C13	−57.41 (19)
C8—N2—C1—O1	58.28 (19)	N2—C8—C13—C12	177.61 (13)
C14—N2—C1—N1	66.67 (19)	C9—C8—C13—C12	−57.02 (17)
C8—N2—C1—N1	−116.33 (15)	C11—C12—C13—C8	57.10 (19)
C1—N1—C2—C7	136.11 (15)	C1—N2—C14—O2	−175.44 (14)
C1—N1—C2—C3	−101.33 (17)	C8—N2—C14—O2	7.6 (2)
N1—C2—C3—C4	−178.62 (14)	C1—N2—C14—C15	6.9 (2)
C7—C2—C3—C4	−56.4 (2)	C8—N2—C14—C15	−170.02 (13)
C2—C3—C4—C5	56.0 (2)	C16—N3—C15—C19	−0.6 (3)
C3—C4—C5—C6	−55.1 (2)	C16—N3—C15—C14	174.98 (15)
C4—C5—C6—C7	55.2 (2)	O2—C14—C15—N3	−146.42 (16)
N1—C2—C7—C6	178.06 (15)	N2—C14—C15—N3	31.3 (2)
C3—C2—C7—C6	55.8 (2)	O2—C14—C15—C19	29.4 (2)
C5—C6—C7—C2	−55.4 (2)	N2—C14—C15—C19	−152.84 (15)
C14—N2—C8—C9	87.09 (17)	C15—N3—C16—C17	0.5 (3)
C1—N2—C8—C9	−90.08 (17)	N3—C16—C17—C18	0.4 (3)
C14—N2—C8—C13	−147.52 (15)	C16—C17—C18—C19	−1.3 (3)
C1—N2—C8—C13	35.31 (19)	N3—C15—C19—C18	−0.2 (3)
N2—C8—C9—C10	−178.34 (13)	C14—C15—C19—C18	−175.81 (15)
C13—C8—C9—C10	56.45 (17)	C17—C18—C19—C15	1.2 (3)

### Hydrogen-bond geometry (Å, °)

**Table:**

*D*—H···*A*	*D*—H	H···*A*	*D*···*A*	*D*—H···*A*
N1—H1n···O1^i^	0.877 (12)	2.067 (11)	2.908 (3)	160.4 (18)
O1w—H1w···O2	0.84 (2)	1.98 (2)	2.820 (3)	173.7 (19)
O1w—H2w···O1w^ii^	0.84 (3)	1.97 (3)	2.773 (4)	162 (4)
O1w—H3w···O1w^iii^	0.84 (3)	1.98 (3)	2.799 (4)	167 (4)
C17—H17···O1w^iv^	0.95	2.59	3.517 (4)	164
C18—H18···O2^v^	0.95	2.47	3.367 (4)	157

Symmetry codes: (i) *x*, *y*+1, *z*; (ii) −*x*+2, −*y*+1, −*z*+1; (iii) −*x*+2, −*y*, −*z*+1; (iv) *x*, −*y*+1/2, *z*+1/2; (v) −*x*+2, *y*+1/2, −*z*+3/2.
